# Quinine in Continued Fever

**Published:** 1852-08

**Authors:** Wm. M. Bolling

**Affiliations:** Montgomery, Ala.


*Quinine in Continued Fever.* By WM. M. BOLLING, M. D., of Montgomery, Ala.—But although I have never myself been able to cut short by the use of Quinine, an unquestionable case of Typhoid Fever, and although it is now, I believe, pretty generally the impression among such physicians of this section of Alabama as I have conversed with on the subject, that it cannot be so arrested, it is more, whatever my own belief, than I would be willing to assert, that it may not be done. I have never myself given the remedy in Typhoid Fever to the extent indeed that Dr. Fearn did, in the cases in which he succeeded with its use, though I have frequently given it in *mild* cases without this effect, in doses, with which I am in the habit daily of arresting with certainty and at once the most *violent* attacks of the various forms of miasmatic fever.—Either then my doses have been too small, or the disease now called Typhoid Fever among us, is different from the cases which were treated with Quinine successfully by Dr. Fearn, and which he calls Typhus, notwithstanding the striking resemblance between them,—and at all events, in both there is this agreement, that in their symptomology they differ widely from any of our recognized shades of miasmatic fever. Besides the name of Typhus which he gives his cases, Dr. Fearn speaks of the disease as *continued fever.* He does not speak, however, of the post mortem appearances, and notwithstanding the resemblance, I am forced to the belief, that the disease in question was not the one now known among us as Typhoid Fever. I cannot think it possible that this malady, when established in a recognizable form, can be cut short by Quinine. With great partiality for the remedy, and not a little confidence of success, based upon a long and satisfactory use of it in many other diseases, I commenced its administration in Typhoid Fever, and was not less astonished than mortified at the result. Reflecting upon the probable cause of my failure to cut short the disease in its progress, by a remedy from which I expected so much, notwithstanding that I could temporarily control the febrile action, it occurred to me that it might be owing to an inherent tardiness of the reparative process, in the lesion of the intestinal glands. With the view, consequently, of preventing, if possible, this intestinal lesion, by arresting in its incipiency the fever, the *very early* administration of Quinine became a main point in the treatment of the disease with me, till, I think, the experiment was fairly tried.

I have given Quinine in Typhoid Fever, in its various stages, but without observing any difference in its effects, in any way connected with, or growing out of, the period to which the case had extended. I have given it in some cases as soon as the nature of the case was manifest, and n others, at various periods to the termination of the fourth, fifth or sixth week, and still the effect has been the same. I have given it, too, at so early a period, that no one, from the symptoms, could say whether the case was or was not one of Typhoid Fever, and yet the character of the case has been developed and declared after its suspension, and the disease has pursued its ordinary course. Possible exceptions have been mentioned.

As to my doses—in one case I gave twenty grains, repeating it in two hours, and thirty-six grains daily for the two or three succeeding days, in three doses of twelve grains each, at intervals of two hours; always between midnight and day, supposing that the febrile action would be less at this time. In other cases, I have given daily say two doses of 16 grains each, or three doses of 12 grains each, at short intervals, repeated for several days in succession; and again I have kept up its continuous operation by doses of 8 or 10 grains, repeated every six or eight hours for several days; and always the result has been much the same. Nor have I neglected any adjuvant measures with which I was acquainted, calculated to secure its beneficial influence. I have given it in eombination with full doses of Morphia, and I have given it without; I have preceded its administration, where the state of the bowels would admit of it, with small doses of blue mass; I have used the warm foot bath and warm drinks; I have used tepid or cold ablution of the entire surface, and cold drinks.—*N. O. Med. Jour.*

